# Gate Control of Superconductivity in Mesoscopic All-Metallic Devices

**DOI:** 10.3390/ma14051243

**Published:** 2021-03-05

**Authors:** Claudio Puglia, Giorgio De Simoni, Francesco Giazotto

**Affiliations:** 1 Department of Physics, University of Pisa, Largo Pontecorvo 3, I-56127 Pisa, Italy; 2 NEST, Instituto Nanoscienze-CNR and Scuola Normale Superiore, I-56127 Pisa, Italy; giorgio.desimoni@nano.cnr.it (G.D.S.); francesco.giazotto@sns.it (F.G.)

**Keywords:** superconductivity, Josephson effect, gate control

## Abstract

The possibility to tune, through the application of a control gate voltage, the superconducting properties of mesoscopic devices based on Bardeen–Cooper–Schrieffer metals was recently demonstrated. Despite the extensive experimental evidence obtained on different materials and geometries, a description of the microscopic mechanism at the basis of such an unconventional effect has not been provided yet. This work discusses the technological potential of gate control of superconductivity in metallic superconductors and revises the experimental results, which provide information regarding a possible thermal origin of the effect: first, we review experiments performed on high-critical-temperature elemental superconductors (niobium and vanadium) and show how devices based on these materials can be exploited to realize basic electronic tools, such as a half-wave rectifier. Second, we discuss the origin of the gating effect by showing gate-driven suppression of the supercurrent in a suspended titanium wire and by providing a comparison between thermal and electric switching current probability distributions. Furthermore, we discuss the cold field-emission of electrons from the gate employing finite element simulations and compare the results with experimental data. In our view, the presented data provide a strong indication regarding the unlikelihood of the thermal origin of the gating effect.

## 1. Introduction

In the last two years, the impact of gate voltage on the superconducting properties of Bardeen–Cooper–Schrieffer (BCS) [1] elemental superconductors has been investigated [2,3,4,5,6,7,8]. In these studies, the authors analyzed the effect of electrostatic gating, generating electric fields reaching the order of $10^{8}$ V/m and, at the same time, creating negligible variations in the surface charge carrier concentration. Although an increase in the critical temperature of a superconducting NbN wire was reported [8], the majority of works in this field show ambipolar suppression of supercurrent, e.g., in all-metallic superconductor wires [2], nano-constriction Josephson junctions (JJs) [3,4], fully metallic Superconducting Quantum Interference Devices (SQUID) [6], and proximity nanojunctions [9]. Such an unconventional gating effect in BCS superconductor systems is the first step in the realization of easy fabrication and high-scalable technologies in both environments of classic superconducting electronics and quantum computing. The purpose of this review is to cover recent advances in the control of superconducting properties in mesoscopic structures via the application of a control gate voltage. For such an effect, a fulfilling microscopic theory has not been provided yet. Indeed, it is not possible to take into account experimental observations through the conventional BCS framework, in which the superconducting properties are negligibly affected by electric fields [10]. Although some theories have been proposed, including surface nucleation and pinning of Abrikosov vortices [8,11,12,13], the electric field-driven Rashba orbital polarization [14,15,16,17], and the gate-driven Schwinger excitation of quasiparticles from the BCS vacuum [18,19,20], they have not been experimentally verified yet. The injection of high-energy field-emitted cold-electrons into the weak-link was also hypothesized to be at the origin of the gating effect [21,22]. Nevertheless, even in the presence of the latter mechanism, several experimental results are not compatible with a mere power injection, resulting in overheating of the superconductor [2,3,23,24].

This article is organized as follows: Section 2 displays evidence of a gate-driven supercurrent suppression in vanadium and niobium Dayem bridges (DBs). Moreover, different technological implementations based on these materials are presented. In the same section, two further topics are faced. The former is modification of the switching current probability distribution as a function of the gate voltage. The latter is influence of the substrate on the gating effect in titanium weak-links. Section 3 analyzes the evidence against a thermal origin of the supercurrent suppression. Finally, Section 4 provides a summary of the results presented in this review, reiterating the main achievements and proposing new experiments to increase the understanding of the gating effect.

## 2. Gate-Driven Supercurrent Suppression in Nb and V Nanojunctions

In this section, we present a series of experiments, performed on niobium and vanadium superconducting weak-link devices, aimed at extending the range of materials suitable for gated-superconductor applications for elemental superconductors with a critical temperature higher than the liquid helium temperature ∼4.2 K. The presented results demonstrate the possibility to implement gate-controlled all-metallic superconducting electronics [25] compatible with industrial standards.

### 2.1. Niobium Gate-Controlled Transistor

**Figure 1:**
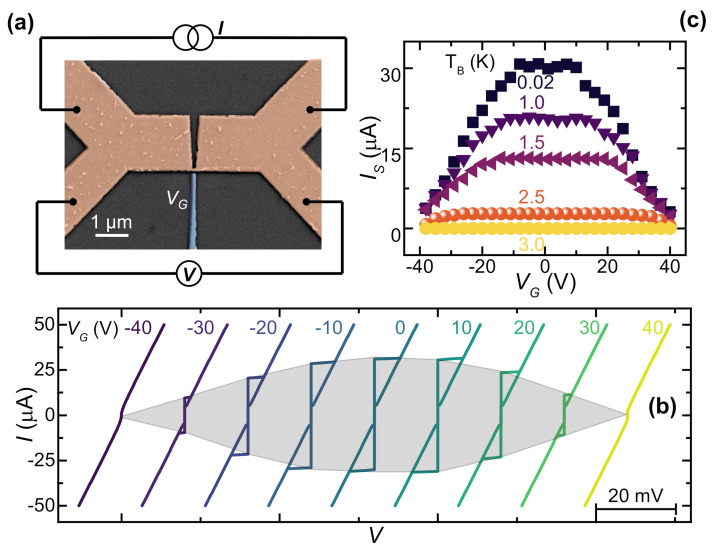
(**a**) Pseudo-color scanning electron micrograph (SEM) of a typical niobium gated transistor with the bias scheme. The weak-link and the wire are in false-colored orange, and the gate is in blue. (**b**) *I* vs. *V* curves for select gate voltages $V_{G}$ at a bath temperature of 20 mK. The curves are horizontally offset for clarity. Bipolar suppression of the $I_{S}$ is visible as $\left|V_{G}\right|$ increases. (**c**) $I_{S}$ vs. $V_{G}$ for several bath temperatures *T* ranging between 20 mK and 3 K. $I_{S}$ values were collected by measuring 50 repetitions of the *I* vs. *V* characteristics.

All-metallic supercurrent transistors consist of a superconducting mesoscopic channel realized with BCS metals, equipped with gate electrodes lithographically fabricated at a distance of a few nanometers from the channel. The gate electrode is polarized through the application of either positive or negative control gate voltage. Niobium gate-controlled transistors typically consist of an 8-μm-long, 2.5-μm-wide wire interrupted by a 50-nm-wide, 120-nm-long constriction. Aligned with the DB weak-link, a co-planar, 60-nm-far, 80-nm-wide metallic gate was realized. The thin film was deposited on a sapphire Al${}_{2}$O${}_{3}$ substrate via DC magneto-sputter deposition of a 10/40-nm-thick Ti/Nb bilayer. The former metal was necessary to increase the adhesion and the mechanical strength of the metallic film. A pseudo-color scanning electron micrograph is shown in Figure 1a.

The device shows a Dayem bridge normal state resistance $R_{DB}≃30\;\Omega$ and a critical temperature $T_{DB}≃3$ K [26]. On the other hand, the niobium banks inherit the critical temperature of the pristine thin film $T_{Nb}≃7.9$ K [26]. The smaller critical temperature of the Dayem bridge is due to its lateral size, which is comparable with the niobium coherence length [27,28]. $T_{Nb}$, instead, is about 80% of the conventional critical temperature for Nb because of the proximity effect of the adhesion titanium layer. The conventional dissipation-less transport is highlighted by plotting the current–voltage *I* vs. *V* characteristics measured at a bath temperature T=20 mK and a gate voltage $V_{G}=0$ V, as shown in Figure 1b. The Dayem bridge switching current is $I_{S}≃30\;$μA. The forward and backward *I* vs. *V* characteristics show the conventional hysteretic behavior due to heating induced by the current bias when switching from the normal to the superconducting state (at the retrapping current $I_{R}$) [29,30].

Suppression of the switching current via the application of a gate voltage was demonstrated by measuring the *I* vs. *V* characteristics as a function of $V_{G}$ from −40 to 40 V at a bath temperature of T=20 mK. Figure 1b displays V(I) curves at several gate voltages. A shadow grey area is drawn to underline the suppression region. The quenching of the supercurrent is symmetric for $V_{G}⟶-V_{G}$ for bath temperatures between 20 mK and 3 K, as shown in Figure 1c. As reported in conventional experiments [2,3,4,9], widening of the plateau in which the gate voltage is not effective is visible as the temperature rises. The suppression of the supercurrent can be observed up to a temperature of 3 K, with complete suppression at $\left|V_{G}\right|=40$ V for T>2 K. Notably, the suppression of $I_{S}$ is visible up to a temperature of 3 K because the gating affects a localized region of the superconductor around the constriction [24]. When that region switches to the normal state, the screening of the metallic system in the normal state does not allow us to observe the quenching effect.

#### 2.1.1. Rectification Properties

**Figure 2:**
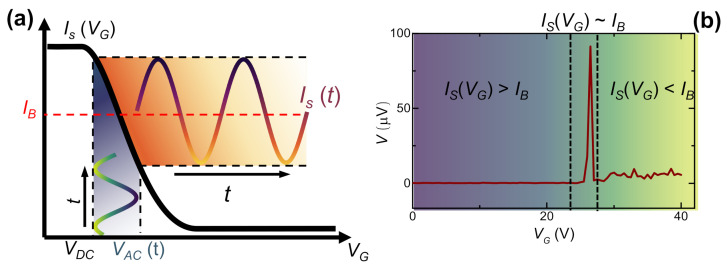
(**a**) Operation scheme of the niobium-based half-wave rectifier. The current bias is represented by the horizontal red dashed line in the $I_{S}\left(V_{G}\right)$ graph. The time-dependent gate voltage (green to blues curve) is composed of an AC component $V_{AC}$ added to a DC bias $V_{DC}$. The effect of the gating provides a time-dependent switching current $I_{S}\left(t\right)$ (purple to yellow line) able to rectify the gate voltage signal. (**b**) *V* vs. $V_{G}$ characteristic of the Josephson junction (JJ) measured with a four-probe technique with a lock-in amplifier. The reference signal of the lock-in is $V_{AC}$, and the bias current $I_{B}$ was set to 2.5μA. *V*. The signal is almost zero until $I_{S}\left(V_{G}\right)<I_{B}$; then, a peak arises due to rectification of the $V_{G}$ signal.

Based on the peculiar shape of the *R* vs. $V_{G}$ characteristic [26], it is possible to implement a superconducting diode scheme. In particular, the sharpness of the super-to-normal transition can be exploited to rectify an alternate voltage $V_{AC}$ applied to the gate electrode while the weak-link is current-biased. In this configuration, a sinusoidal gate voltage is transformed into a square wave voltage-drop across the junction. Such a peculiar system response is shown in Figure 2a. The gate voltage signal is the sum of $V_{AC}$ and of a DC pre-bias voltage used to define a switching current working range $I_{S}\left(V_{G}^{max}\right)<I_{B}<I_{S}\left(V_{G}^{min}\right)$. The oscillation of $I_{S}$ above and below $I_{B}$ results in a periodic normal-to-super and super-to-normal transition that generates a time-dependent voltage-drop V(t) across the junction. The output signal maintains the same periodicity of $V_{AC}$ with a duty cycle given by the time at which $I_{S}<I_{B}$.

Notably, the output voltage depends directly on the amplitude of the AC input signal thanks to the behavior of the *R* vs. $V_{G}$ characteristic. In the configuration shown in Figure 2, our system acts as a half-wave rectifier that could be used in a vast range of devices such as diodes and detectors. In the next paragraph, further evidence of the rectification properties of such systems is provided, with emphases on the versatility of the technology.

### 2.2. Vanadium Gate-Controlled Transistor

**Figure 3:**
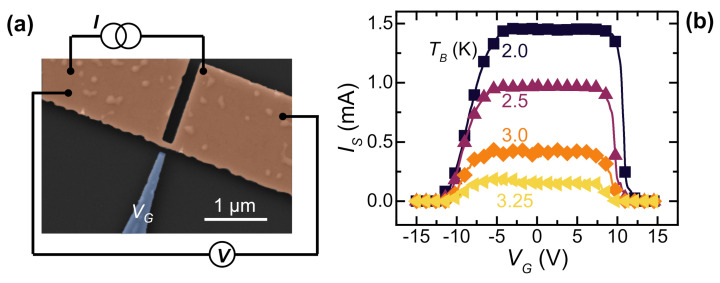
(**a**) Pseudo-color SEM of a representative vanadium-gated device. The weak-link and the wire are colored in orange, and the gate is in blue. (**b**) $I_{S}$ vs. $V_{G}$ curves for different bath temperatures ranging from 2.0 to 3.3 K. The data were computed by averaging 25 acquisitions of $I_{S}$.

The vanadium gate-controlled transistors typically consist of a planar 60-nm-tick, 160-nm-long, 90-nm-wide weak-link with a 70-nm-far, 120-nm-wide gate aligned to the weak-link. The exploited bridge geometry is similar to those already discussed for Nb-based devices. The device fabrication was performed on a silicon/silicon oxide (Si/SiO${}_{2}$) substrate by means of a single-step electron beam lithography followed by an e-beam V evaporation performed at a rate of 0.36 nm/s in an ultra-high vacuum chamber. Figure 3a displays the pseudo-color SEM of a representative vanadium-gated device.

The device shows a normal state resistance $R_{N}≃106\;\Omega$, a switching current at 2 K of $I_{S}=1.42$ mA, and a critical temperature $T_{C}≃3.6$ K [31].

Suppression of the switching current as a function of the gate voltage was demonstrated by measuring the $I_{S}$ vs. $V_{G}$ characteristics. Figure 3b shows bilateral suppression of the supercurrent down to total quenching for $|V_{G}|≃10$ V in a range of bath temperature from 2 to 3.2 K. Notably, the sharper suppression of $I_{S}$ observed for positive values of the gate voltage is in contrast with a possible cold field-emission origin of the quenching effect. Indeed, the device geometry could facilitate electron extraction from the gate that occurs at negative gate bias values [21,22]. This consideration is deeply discussed in Section 3.

#### 2.2.1. Half-Wave Rectifier

**Figure 4:**
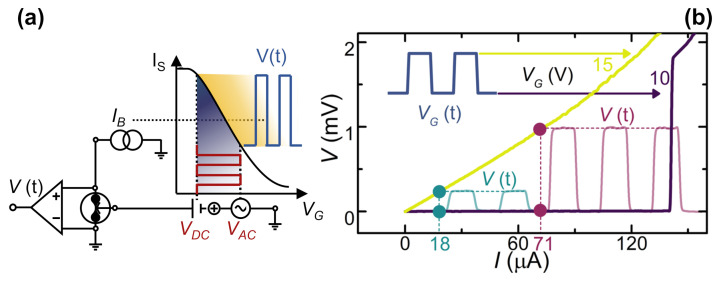
(**a**) Bias scheme for AC measurements. The gate voltage is generated by adding DC $V_{DC}$ and AC $V_{AC}$ arbitrary waveform voltages. The ADC/DAC board that provides the AC signal performs real-time measurements of *V*. (**b**) Voltage *V* vs. current *I* characteristics for different values of $V_{G}$ (yellow and purple curves). The dot couples show the operation points of the system for two different bias currents $I_{B}=18$, 71μA. $V_{G}$ vs. time *t* is the excitation signal (blue curve) that was realized by adding a DC voltage $V_{DC}=10$ V and an AC square-wave voltage with amplitude $V_{AC}=5$ V. Time-dependent *V* for different current biases are drawn in correspondence with the operation points. The measurements were performed at T=3 K.

Time-resolved characterization of the device was carried out using both sinusoidal and square-wave gate voltages. Figure 4a shows the bias scheme of the measurement setup consisting of a DC bias current, a DC voltage generator, and an Analog to Digital Converter/Digital to Analog Converter ADC/DAC digital board providing the AC gate voltage signal. The latter voltage generators provides a $V_{G}\left(t\right)=V_{DC}+V_{AC}\left(t\right)$ gate signal, setting the right operation point in the parameters space (see Figure 4a).

The measured voltage-drop signal V(t) across the junction vs. the time-dependent $V_{G}\left(t\right)$ results in a zero-signal (low-state) when $I_{S}\left[V_{G}\left(t\right)\right]>I_{B}$ (superconducting state). By contrast, when $I_{S}\left[V_{G}\left(t\right)\right]<I_{B}$, the junction switches to the normal state and a finite voltage-drop is built across the DB (high value). We measured the response of the system to a transistor-transistor logic-like (TTL) square-wave excitation consisting of a $V_{DC}=10$ V bias added to a $V_{AC}=5$ V square wave signal with frequencies up to ∼50 Hz, shown in Figure 4b.

The low and high states are highlighted on top of the *I* vs. *V* characteristics (obtained for $V_{G}$ ranging from 10 to 15 V) with dots of the same color in Figure 4b. The V(t) signal resulting from $V_{G}\left(t\right)$ excitation is shown in Figure 4b for two current bias ($I_{S}=18,\;71\;$μA). We note that the output voltage is proportional to the bias current. It is worth emphasizing again that V(t) maintains the shape of the input voltage signal with frequencies, in principle, limited only by $f_{\Delta }$ [30,32].

**Figure 5:**
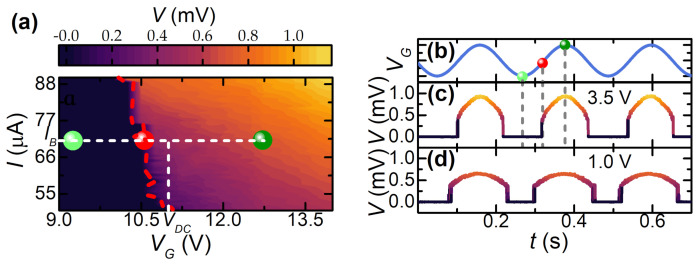
(**a**) Color-plot of *V* vs. $V_{G}$ (x-axis) and *I* (y-axis). From left to right, the three round symbols show the zero-resistance gate voltage value (light green), the super-to-normal transition (red), and the maxima of both $V_{G}$ and *V* (dark green). The dashed red curve represents the $I_{S}$ vs. $V_{G}$ characteristic. (**b**) Time-dependent $V_{G}\left(t\right)$ obtained by adding a DC voltage $V_{DC}=11$ V and an AC sine wave voltage $V_{AC}$. (**c**,**d**) Time-dependent V(t) for $V_{AC}=\;3.5$ V (**c**) and $V_{AC}=1.0$ V (**d**). The color-map is the same as in panel (**a**). All these measurements were performed at T=3 K.

Finally, we show the response of the system to a sinusoidal gate voltage signal. The measurement setup is the same as the square-wave characterization shown in Figure 4a. The excitation consists of a $V_{AC}$ sine-wave with amplitude ranging from 1.0 to 3.5 V summed with a $V_{DC}=11$ V voltage bias, shown in Figure 5b. The bias current for this experiment was chosen to be $I_{B}=72\;$μA to have a sharp super-to-normal transition and a linear dependence between *R* and $V_{G}$. The continuous variation in the gate voltage provides continuous variation in junction resistance accordingly with the $R(V_{G})$ curves [31]. Due to the former behavior, the system is in the non-dissipative state for $I_{S}\left[V_{G}\left(t\right)\right]>I_{B}$ and a voltage-drop across the DB arises when the condition $I_{S}\left[V_{G}\left(t\right)\right]≃I_{B}$ is satisfied. When $I_{S}\left[V_{G}\left(t\right)\right]<I_{B}$, the voltage-drop increases due to gate-driven evolution of the DB resistance and eventually saturates at the asymptotic value of the normal-state resistance. Figure 5a shows the voltage-drop across the junction as a function of both the bias current and the gate voltage. The transition edge is highlighted by a dashed red line. The working point set by $I_{B}$ and $V_{DC}$ is shown by white dashed lines. The time-resolved V(t)s for $V_{AC}$ equal to 1.0 and 3.5 V are reported in Figure 5c,d. Notably, the rectification threshold and the portion of the input signal rectified can be selected by setting both $I_{B}$ and $V_{AC}$.

The former characteristics here, typical of a half-wave rectifier, are realized for the first time by exploiting an all-metallic, gated superconducting Dayem bridge. We speculate that the described rectifying behavior can be suitably exploited to rectify incoming radiations coupled to the gate through an antenna, realizing a gate-controlled version of a transition edge sensor [33,34,35]. The rectifier is based on superconducting field-effect transistor FET technology that is controlled via the application of a gate voltage in a similar way to conventional complementary metal–oxide–semiconductor CMOS technology, making the standards perfectly compatible. In the absence of a complete high-frequency characterization, we assume an operational frequency of the order of the gap. For example, Nb and NbN promise to be exploited to realize a transistor with a switching frequency larger than 700 GHz. This device could operate in an extremely wide frequency range, spanning from below 1 GHz to about 1 THz. This interval is particularly relevant for both technological applications and fundamental research (e.g., in astrophysics for cosmic microwave background detection).

#### 2.2.2. Amplification Properties

The vanadium Dayem bridge, thanks to its peculiar $R(V_{G})$ characteristics, is suitable for the realization of an amplifier. The gain parameter of a DB transistor is conveniently defined as the ratio between peak-to-peak amplitudes of gate voltage input and the output voltage-drop across the junction $g=\frac{V_{out}}{V_{in}}$. For our system, $V_{out}=R\left(V_{G}\right)I_{B}$ is the voltage-drop across the junction and is directly proportional to the resistance and the current bias. $V_{in}$ is defined as the ratio between the width of the switching current probability distribution (SCPD) [23] and the transconductance $\tau =\frac{dI_{S}}{dV_{G}}$. For the devices taken into account in this section, g∼7 with a typical power consumption of ∼40 nW. It is worth highlighting, on the one hand, that *g* is of the same order of magnitude as the conventional semiconductor cold amplifier [36,37]. Such a result, on the other hand, was obtained with a power usage smaller than a factor of a thousand compared to the typical semiconducting counterpart. Furthermore, using *N* rectifiers connected in series, feeding the gate electrode of the *N*th rectifier with the output voltage of the (N−1)th one, a total gain equal to $g^{N}$ can be obtained.

The possibility to tune the supercurrent via electrostatic gating paves the way for a wide range of applications. Indeed, gate-controlled devices could be exploited to realize tunable magnetometers [38,39] and heat control systems [40,41]. Furthermore, by exploiting the gating effect, a voltage-controlled version of the nanocryotron [42,43] can be implemented. The latter is a three-terminal superconducting device in which a localized switching-current suppression (triggered by injecting a control-current that generates a hotspot by Joule heating) destroys the superconducting characteristics of the nearby material. The gated version of the nanocryotron, the so-called (EF-Tron) [6], is implemented in parallel to a resistor and a gated superconductor. Differently from current-driven devices [43,44], the EF-Tron is expected not to be limited by the characteristic time scale of thermal effects, which does not allow us to use signals with frequencies larger than about hundreds of MHz at cryogenic temperatures [29]. In this view, it is worth discussing the role of an eventual direct power injection into the gated device (driven, e.g., by a gate-superconductor leakage current) that could produce an increase in the electronic temperature, detrimental for device performance.

## 3. Nonthermal Origin of Supercurrent Suppression in Gated All-Metallic Superconducting Devices

### 3.1. SCPDs in a Titanium Gate-Controlled Transistor

In a current biased Josephson junction (JJ), the super-to-normal transition for fixed values of external parameters, e.g., temperature, and electric and magnetic fields, is triggered by phase slips. Such events, where the amplitude of the order parameter coherently fluctuates to zero at some point, recover with a different winding number, resulting in local random 2π jumps of the macroscopic quantum phase ϕ [45]. The accidental nature of such events leads to a non-univocal definition of the switching current, the value of which is distributed according to the switching current probability distribution (SCPD). The investigation of the SCPD of a JJ is, therefore, exploited to probe the dynamics of the phase slips. Here, we discuss an experiment where a well-established technique is adopted to probe the impact of gate voltage on the number of phase slip events in gate-controlled fully metallic titanium-based Dayem bridges with the conventional theory [46,47,48].

**Figure 6:**
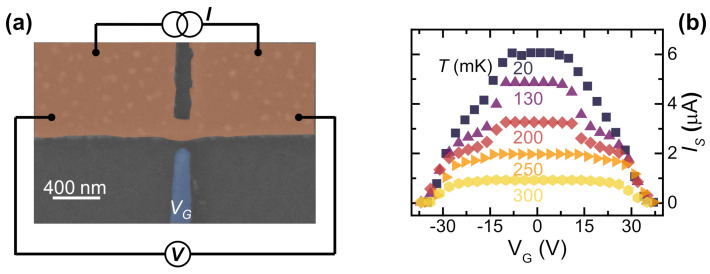
(**a**) Pseudo-color (SEM) and bias scheme of a representative Ti gate-controlled transistor. The superconducting wire and the Dayem bridge constriction are colored in orange, and the gate electrode is in blue. (**b**) $I_{S}$ vs. $V_{G}$ characteristics at select bath temperatures ranging from 20 to 300 mK. Data are the result of the average of 50 acquisitions of $I_{S}$.

The device chosen to study the evolution of the SCPD under gating action consists of a titanium Dayem bridge. Such JJs consist of 30-nm-thick, 10-μm-long, 2.5-μm-wide wires interrupted by a constriction. This 30-nm-thick, 150-nm-long, 120-nm-wide narrow structure was aligned with a planar, 80-nm-far, 140-nm-wide metallic gate. The sample was fabricated using single-step electron beam lithography on a sapphire (Al${}_{2}$O${}_{3}$) crystal wafer. The Ti layer was deposited at a rate of 1.2 nm/s. Figure 6a shows a pseudo-color scanning electron micrograph.

The device shows a normal state resistance $R_{N}≃550\;\Omega$, a switching current at 20 mK of $I_{S}=6.0\;$μA, and a critical temperature $T_{C}≃310$ mK [23].

The dependence of $I_{S}$[2,3,4,5,6,9,23,26,31] on the gate voltage is shown to acquire the $I_{S}$ vs. $V_{G}$ characteristics as a function of bath temperature. Figure 6b shows that the supercurrent vanishes for $\left|V_{G}\right|\;≃\;34$ V and that such a value appears to be independent from the bath temperature. By increasing the values of the temperature, $I_{S}^{0}=I_{S}\left(V_{G}=0\right)$ lowers and $I_{S}$ is unaffected by the gate voltage for a larger range of $V_{G}$. This latter behavior resembles the results obtained on Ti and Al superconducting FETs [2,3].

To characterize the effect of temperature on superconducting Dayem-bridge JJs, SCPDs were measured at different values of thermal bath temperature. The distributions were reconstructed by drawing a histogram based on $10^{5}$ switching current acquisitions for each value of the bath temperature *T*.

**Figure 7:**
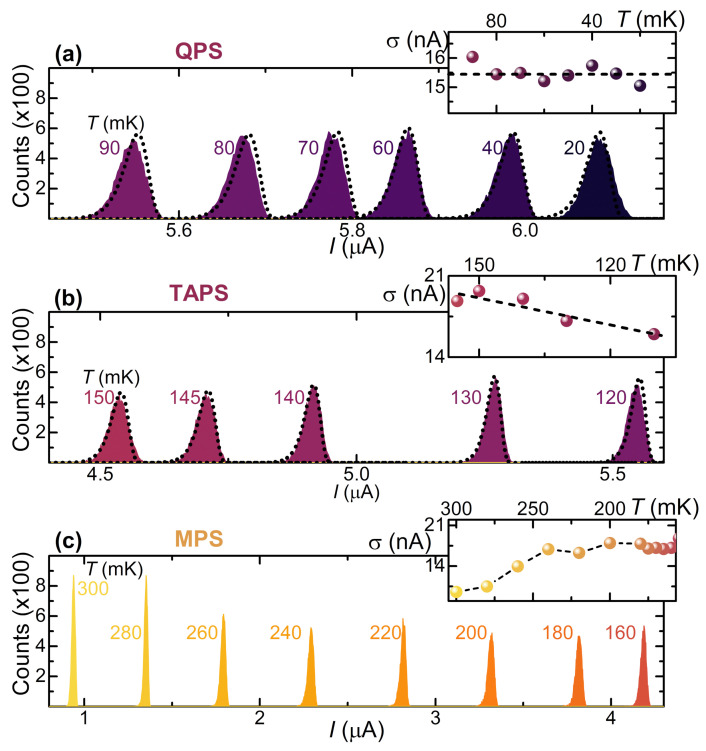
(**a**) Switching current probability distributions (SCPDs) vs. *I* acquired at select bath temperatures from 20 to 90 mK in the Quantum Phase Slip (QPS) regime. The best fit curves are represented with dotted line. The inset shows σ vs. *T* of the regime. (**b**) SCPDs vs. *I* obtained at different temperatures from 120 to 150 mK in the Thermal Activated Phase Slip (TAPS) regime. The best fit curves are represented with a dotted line. The inset shows σ vs. *T* of the regime. (**c**) SCPDs vs. *I* obtained at different temperatures from 160 to 300 mK in the Multiple Phase Slip (MPS) regime. The inset shows σ vs. *T* of the regime. For each SCPD, the total sampling number of $I_{S}$ is $10^{5}$. The crossover temperatures $T_{Q}≃110$ mK and $T_{M}≃160$ mK separate the QPS/TAPS and TAPS/MPS regimes, respectively. In all the panels, the temperature increases from right to left.

Figure 7 shows the evolution of these so-called thermal SCPDs in a temperature range between 20 and 300 mK. The dependence of the shape of thermal SCPDs is analyzed through the conventional Kurkijärvi–Fulton–Dunkleberger (KFD) theory [46,49] with a fit procedure [23]. First, the different phase slip regimes were identified thanks to the evolution of distribution standard deviation σ as a function of *T*. The Figure 7 insets show the expected behavior of the σ vs. *T* curve in the three different phase slips regimes, which is flat at low temperatures in the Quantum Phase Slip (QPS) regime [50,51,52], linear as a function of the temperature *T* in the Thermal Activated Phase Slip (TAPS) regime [53], and decreasing for the Multiple Phase Slip (MPS) regime as *T* increases [45,46,47,49,54,55,56,57]. The temperature $T_{Q}$, separating the QPS and the TAPS regime, occurs at about T≃110 mK, while the crossover between TAPS and MPS regimes appears for $T_{M}≃160$ mK.

**Figure 8:**
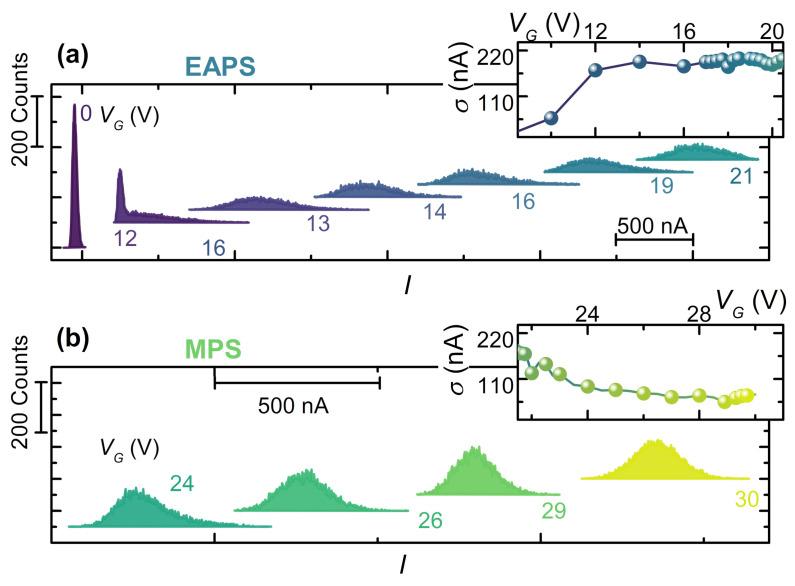
(**a**) SCPDs vs. *I* at select gate voltages from 0 V to 21 V in the Electric Activated Phase Slip (EAPS) regime. The inset shows standard deviation σ of SCPDs vs. gate voltage $V_{G}$ in the EAPS regime. (**b**) SCPD vs. *I* at different gate voltage values from 24 V to 30 V in the MPS regime. The inset shows standard deviation σ of SCPDs vs. gate voltage $V_{G}$ in the MPS regime. For each distribution, the total number of $I_{S}$ acquisitions is $10^{5}$. The curves are vertically offset for clarity. The crossover voltages are $V_{Q}≃8$ V and $V_{E}≃14$ V.

Although these devices show the conventional behavior of phase slip dynamics as the temperature *T* varies, the gate voltage drives the junction in a different regime. Figure 8a shows vertically shifted SCPDs collected for several values of the gate voltage at T=20 mK. In particular, the SCPDs overlap for $V_{G}<8$ V whereas a low-current “tail” is observed for $8<V_{G}<14$ V. In addition, the distributions greatly widen for $14<V_{G}<24$ V, and for high gate voltage values, i.e., $V_{G}>24$ V, the SCPDs turns out to be narrow. In this electrostatically driven scenario, the σ vs. $V_{G}$ curve takes the place of the conventional σ vs. *T* characteristic. As shown in Figure 8b, the $\sigma (V_{G})$ curve exhibits a region of constant standard deviation, thereby highlighting a marginal contribution of the gating effect to the number of phase slip events for low $V_{G}$ values. Such behavior seems to be similar to the conventional QPS regime. Therefore, we identify a crossover gate voltage $V_{Q}≃8$ V between the former and the Electric Activated Phase Slip (EAPS) regime, where the σ grows to ∼200 nA as the gate voltage increases. Notably, σ starts to increase at the same voltage at which the switching current begins to be affected by the electric field. Such evidence seems to suggest that, whatever the origin of $I_{S}$ suppression, the latter is associated with a corresponding increase in phase slip events. Finally, for higher values of the gate voltage (i.e., $V_{G}>V_{E}≃14$ V), σ decreases and saturates to ∼75 nA.

**Figure 9:**
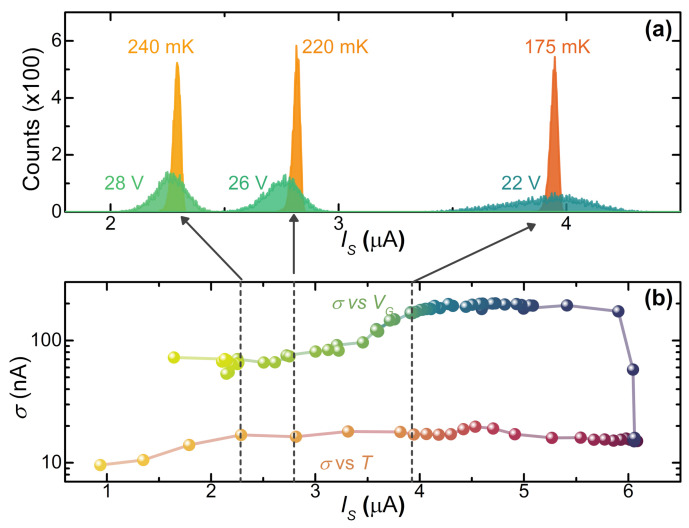
(**a**) $I_{S}$-matched distributions. Red and orange distributions were acquired for a negligible electric field at $V_{G}=0$ V at select bath temperatures, whereas blue and green distributions were measured at T=20 mK for different gate voltage values. The values of $I_{S}$ are, respectively, from left to right 2.2,2.8, and 4.0μA. (**b**) Comparison between the σ vs. $I_{S}$ characteristic obtained for thermal- and electric-driven distributions at $V_{G}=0$ V (lower curve) and T=20 mK (upper curve) respectively.

The starkly different behavior between thermal and electric SCPDs is displayed in Figure 9a, where three $I_{S}$-matched couples of thermal and electric distributions are plotted in the same graph for comparison. The $I_{S}$-matched SCPDs display remarkably different widths and general shapes, an evolution which likely stems from an gate-driven non-equilibrium state induced in the weak-link. In particular, the gate voltage seems to increase the phase fluctuation in the system, allowing for a switching event in a larger current range. Concerning the standard deviation of the distributions, on the one hand, the comparison between σ vs. $I_{S}$ curves extracted from the two thermal and electric SCPDs series, shown in Figure 9b, displays a qualitatively similar behavior. On the other hand, the electric-driven SCPDs present σ on average around one order of magnitude larger.

Indeed, if we assume that the voltage-driven broadening of the SCPD is due to an increase in the electronic temperature, e.g., a trivial Joule heating due to a gate-DB current, we run into the absurdity of obtaining an electronic temperature higher than the critical temperature of the superconductor [29,45]. This observation reflects the impossibility to fit the gate-driven SCPDs with a conventional KFD transform since the resulting fit parameters would be nonphysical. Therefore, on the one hand, these data demonstrate a strong link between phase slip events and electric field and, on the other hand, they suggest a nonthermal origin of the switching current suppression: the action of the gate voltage drives the DB in a state in which the description is incompatible with that of a superconductor heated through a voltage-driven power injection at a thermal steady state with an electronic temperature higher than that of the thermal bath.

### 3.2. Suspended Titanium Gate-Controlled Transistor

We have shown that it is not possible to describe the modification of SCPDs as a consequence of trivial overheating. In other words, the effect of the gate is unlikely that of driving the superconductor into a higher electronic-temperature steady-state by mere power injection (driven by current injection). To further investigate the role of a possible injection of current between the gate and superconducting channel, fully suspended, gated superconducting nanobridges were tested. In conventional gated devices, two gate-channel charge transport mechanisms might be present: the diffusive current injection through the substrate and the ballistic emission of cold-electrons (CFE) across the vacuum. The suspended geometry permits us to exclude leaving the CFE as the only possible charge transport mechanism.

**Figure 10:**
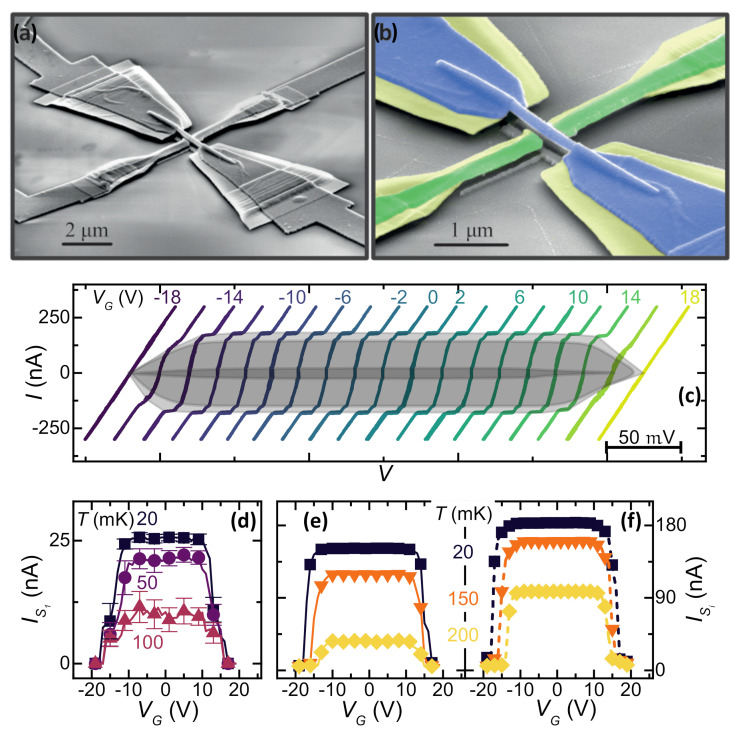
(**a**,**b**) SEMs of the suspended titanium transistor (original picture and pseudo-color). (**c**) Back and forth current *I* vs. *V* characteristics for select values of $V_{G}$ measured at a bath temperature of T=20 mK. The characteristics are horizontally shifted for clarity. Grey colored regions highlight the gate-induced evolution of $I_{S_{1}}$, $I_{S_{2}}$, and $I_{S_{3}}$. (**d**–**f**) The $V_{G}$ dependence of the switching currents of $I_{S_{1}}$, $I_{S_{2}}$, and $I_{S_{3}}$, respectively.

This experiment was performed on titanium-gated suspended wires. The devices consist of 70 nm thick and 1.7 μm long Ti nanobridges deposited on top of an undoped 130-nm-suspended crystalline InAs [58,59] nanowire (NW) realized by chemical beam epitaxy. The NWs were deposited onto a layer of Poly-methyl-methacrylate PMMA spin-coated and baked on top of a Si/SiO${}_{2}$ substrate. Then, the resist were exposed via electron beam lithography (EBL) (5000 μC/cm${}^{2}$ at 10 keV) to cross-link the PMMA underneath the ends of the nanowires and to define the pillars that sustain the NWs. Then, InAs NW suspension was achieved, removing the unexposed PMMA thanks to a 10-min acetone bath. The anchoring between the NW and the PMMA pillars was obtained with an aligned EBL and a thermal evaporation of a Ti/Au (5/15 nm) bi-layer. The portion of cross-linked resist uncovered by the Ti/Au shields was then removed with O${}_{2}$ dry etching. Such a step results in a large undercut along the Au pad edges that prevents short circuits among the wire and the gate electrodes after the last titanium evaporation. Finally, the last EBL step allows us to state that the final titanium wire electron beam evaporates at a rate of about 1.2 nm/s. Figure 10a,b show scanning electron micrographs of a typical device.

Such a suspended nanojunction shows four different superconducting transitions [24] that can be interpreted as the switch of the superconducting banks for $I_{B}≃1.8\;$μA and of the series of three junctions with switching currents at, respectively, $I_{S_{1}}≃25$ nA, $I_{S_{2}}≃$ 150 nA, and $I_{S_{3}}≃180$ nA. The geometry of the junction and the multi-step fabrication technique induced the existence of such a series of three junctions in the nanobridge due to inhomogeneities of the titanium layer thickness deposited on top of an InAs nanowire. Moreover, the switching current difference resides in the variation of the cross section of a Ti film coating the wire and on the inhomogeneous anti-proximization effect of the superconducting film due to the bottom gold layer.

The *I* vs. *V* shifted characteristics at select gate voltages from −20 to 20 V of the bridge are shown in Figure 10c at a temperature of 20 mK. Figure 10d–f shows the evolution of the switching currents $I_{S_{i}}$ of the three junctions as a function of the voltage gate, extracted from the *I* vs. *V* curves measured at several *T*.

Notably, as the temperature increases, the gate voltage range for which $I_{S}$ is unaffected shrinks. The former results are in opposition with previous experiments [2,3,4] on the subject. We attribute this different behavior to the reduction in thermal coupling between the Dayem bridge and substrate compared to systems located on a substrate, whereas in terms of the microscopic origin of the gate-driven effect, the reduction in the switching current appears to be connected to a considerable increase in quasi-particle excitations in the superconducting system [21,23]. Such enhancements seem to be more efficient in suspended devices, in which the relaxation of quasiparticles via electron–phonon interactions is greatly reduced compared to conventional systems. These results demonstrate that the presence of an interface between the substrate and the superconducting junction is not necessary for the gate effect to occur. This is unequivocal evidence against the hypothesis of a Joule heating origin of supercurrent suppression due to a diffusive current injected into the substrate.

### 3.3. Leakage Current Finite Element Method Simulations

The suspended geometry experiment allows us to exclude the Joule heating as the main origin of quenching of the supercurrent in gated metallic structures. In this framework, the only possible mechanism that allows a current to flow between the gate and the wire is vacuum cold-electron field-emission (CFE). Such an emission is typically due to the presence of an intense electrostatic field between different electrodes [60,61]. To understand the role of a hypothetical current generated via field-emission in $I_{S}$ suppression, the CFE current ($I_{FE}$) can be quantified through 3-D finite-element method simulations run on a system with the same geometry as the suspended titanium gate-controlled transistor and then compared with the measured leakage current $I_{L}$[24]. $I_{FE}$ is computed via numerical integration over the cathode surface, i.e., the gate (wire) for negative (positive) gate voltages and the Fowler–Nordheim (FN) current density generated via the tunnelling effect [60,61]:

$$J_{FE}\left(E,h_{0}\right)=\frac{2.2e^{3}}{8\pi hh_{0}}E^{2}\exp\left[-\frac{8\pi }{2.96he|E|}{\left(2m_{e}\right)}^{1/2}h_{0}^{3/2}\right],$$

where E(x,y,z) is the electric field at the surface of the cathode; $m_{e}$ and *e* are the mass and the charge of the electron, respectively; *h* is Plank’s constant; and $h_{0}=4.3$ eV is the literature titanium work function [62]. The Maxwell equations $E=-\nabla V(x,y,z;V_{G})$ allows us to calculate the electric field, where the electrostatic potential was obtained, exploiting the Poisson equation $\nabla^{2}V\left(x,y,z;V_{G}\right)=0$. The boundary conditions for the Poisson equation were simulated with perfect equipotential conductor boundaries set at V=0 and $V=V_{G}$ for the two electrodes.

**Figure 11:**
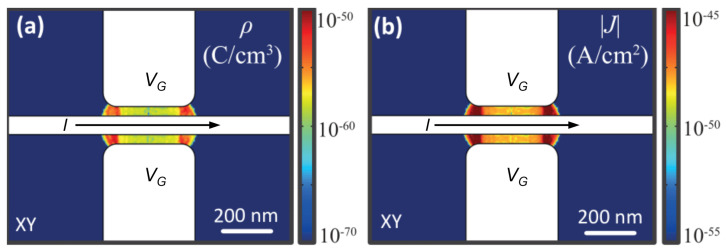
$\left|E(x,y,z)\right|$ and streamlines on the XY (**a**) and YZ (**b**) planes. The simulations were performed with a gate voltage value of $V_{G}=-15$ V. The distribution of the electrostatic field shows that the field effect is confined upon constriction.

The distribution in the space of the electric field module $\left|E\right|$ computed in the entire simulation domain is shown in Figure 11a,b at $V_{G}=15$ V for the top plane and cross-sectional views. The simulations show that the electrostatic field is strictly confined between the titanium constriction and the gate electrode surfaces and that it rapidly decreases elsewhere, without affecting the superconducting banks.

The electric field reaches the maximum intensity of 0.2 GV/m in correspondence with the center of the gate, and it is localized near the side gate surfaces. Moreover, |E(x,y,z)| drops more than one order of magnitude between 500 nm from the lateral edge of the gate electrode. Such a field geometry lets us conclude that the banks are unlikely to be affected by gate voltage.

**Figure 12:**
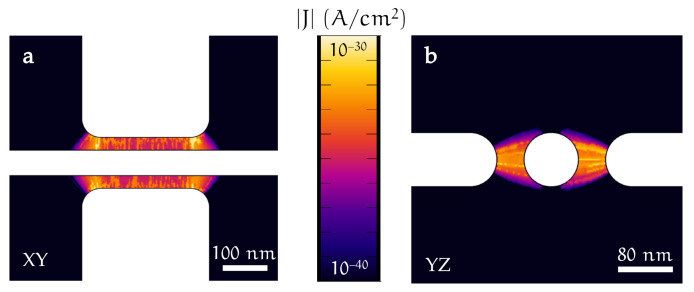
Current density module $\left|J_{FE}\left(x,y,z\right)\right|$ evaluated on XY (**a**) and YZ (**b**) planes. Data were obtained by analyzing the ballistic transport of the electrons through the vacuum from the gate electrode surfaces toward the titanium constriction (and vice versa for opposite values of gate voltage). Here, we set the gate voltage to $V_{G}=-15$ V and the work function equal to the literature value for titanium $\phi_{0}=4.3$ eV. The spatial distribution of the electronic current highlights that the field emitted electrons influence a 500 nm section of the constriction.

By solving the ballistic trajectories of the electron emitted by the electrode, it is possible to compute the current density $|J_{FE}|$ in the region between the gate and the wire. Figure 12a,b shows $|J_{FE}|$ evaluated on the XY and YZ planes. It is worth noticing the extreme localization of the electrons in a region of about 500 nm centered on the electrodes that affects only a small portion of the nanobridge. Such evidence proves that, for cold-emitted electrons, the vast majority of the charge particles emitted/absorbed from the gate are absorbed/emitted from the wire.

**Figure 13:**
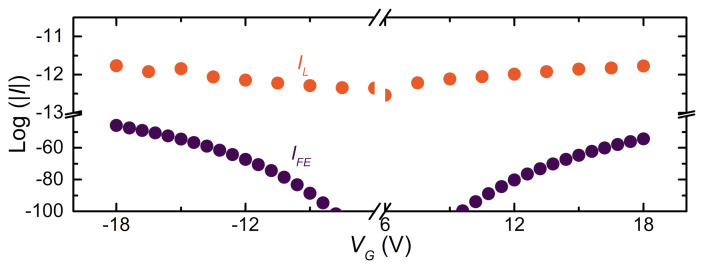
Natural logarithm of $I_{L}$ between the gate electrodes and the constriction at a bath temperature of T=20 mK vs. the gate voltage $V_{G}$ measured on a titanium suspended device (Orange dots). Natural logarithm of $I_{FE}$ between the gate electrodes and the constriction vs. the gate voltage $V_{G}$ computed by integrating the Fowler–Nordheim (FN) current density ($J_{FE}$) with $\phi_{0}=4.3$ eV (blue dots).

Finally, integration of the current density $J_{FE}$ over the surface of the electrodes returns the current $I_{FE}$, a quantity that can be directly compared with the gate-wire current measured in the experiment (shown in Figure 13) [24].

Notably, $I_{FE}$ is at least 20 orders of magnitude smaller than the maximum gate-bridge leakage current experimentally measured. Furthermore, a current of about $10^{-40}$ A corresponds to the emission of a single charged particle every $10^{28}$ years on average, and it is consistent with electrostatic fields that are not strong enough to generate a true cold-emission current. Typically, electrostatic fields of about 1–10 GV/m [63] are requested to generate a proper CFE current, but our results show that the maximum value of |E| is smaller by at least a factor of 10. Moreover, the simulation shows an intrinsic asymmetry of several orders of magnitude for $I_{FE}$ when $V_{G}⟶-V_{G}$ due to the geometry difference between the gate and wire. This seems to suggest that, if the field emitted current was the leading mechanism in determining $I_{S}$ suppression, a strongly asymmetric behavior should be observed for positive and negative gate voltages. Such a feature was never reported in experiments on gated metallic superconductors [2,3,4].

### 3.4. Heating through Single Cold-Electron Field Emission or Absorption

If we admit that a certain number of electrons are emitted or absorbed by the gate and absorbed/emitted by the wire, the expected experimental phenomenology should be different compared to those observed: a single electron with an energy of the order of 10 eV that ballistically reaches the constriction through the vacuum is expected to release its energy, inducing a sudden increment in the system electronic temperature. A straightforward calculation for the electron contribution to the heat capacitance $C_{e}$ of a weak-link in the dissipative state is as follows:

$$C_{e}=\Omega \gamma T_{e}$$

where Ω is the volume occupied by the junction, γ is the Sommerfeld constant for titanium, and $T_{e}$ is the electronic temperature of the system.

The released energy E(V) is proportional to the acceleration voltage *V* between the gate electrode and the Dayem bridge:

$$E\left(V\right)=qV,\;\;\;\;\;\;P\left(t\right)=E\delta \left(t\right)$$

where *q* is the electron charge, δ is Dirac’s delta distribution, and P(t) is the impulse power as a function of time *t*. According to heat transport theory, the evolution of the electronic temperature in the junction is described by the following differential equation [29], where $T_{B}$ is the lattice temperature:

$$C_{e}\frac{\partial T_{e}}{\partial t}=P\left(t\right)⟶T_{e}=\sqrt{\frac{2E}{\Omega \gamma }+T_{B}^{2}}$$

We wish to stress that, by this approach, we obtain the final electronic temperature $T_{e}$, which is an underestimate of its real value in the weak-link since we considered $C_{e}$ to be that of the normal state, which is exponentially larger than in the non-dissipative state because of the energy gap in the density of states [29]. The calculation demonstrates that single electrons with an energy of approximately 30 eV that release their energy into the superconducting system at a bath temperature of 10 mK would increase the electronic temperature to a value 20 times larger than its critical temperature ($T_{C}≃500$ mK). Such a result is evidence that the heat generated from CFE electron absorption in the nanoconstrictions cannot lead the system in an equilibrium condition with well-defined gate-driven $T_{e}$ and $I_{S}$.

### 3.5. Continuous Power Injection

**Figure 14:**
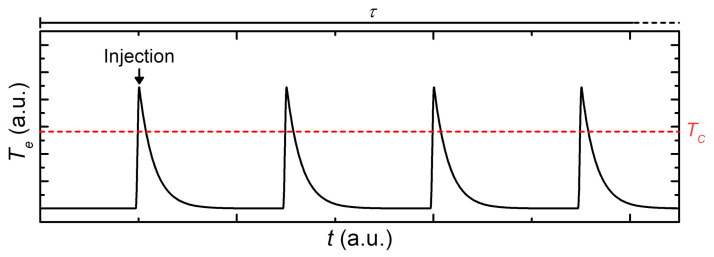
Electronic temperature $T_{e}$ vs. time *t* of a mesoscopic superconducting weak-link that periodically absorbs electrons with an energy of the order of 10 eV. The red horizontal line represents the critical temperature of the superconductor. Each electron starkly increases the electronic temperature of the system, driving it in the normal state. τ is the measurement time.

In principle, we can make the further hypothesis that, due to continuous absorption of highly energetic electrons, the Dayem bridges bounce continuously between its normal and superconducting states, as shown in Figure 14.

In this framework, for each absorbed electron, the system suddenly switches to the normal state. Then, it relaxes to the non-dissipative state in the time given by the electron–phonon interaction, which is, in similar systems, about τ≃1 ns [29], and it is several orders of magnitude lower than the integration time of measurement setup (typically ∼20 ms). In this picture of the effect, during a conventional *V* vs. *I* measurement, for each electron absorption when *I* is below the retrapping current ($I_{R}$), the variation of its resistance is expected to be detected too fast by the measurement instruments. In the opposite situation, when the bias current is larger than $I_{R}$, each time an electron is absorbed, the constriction is driven to the normal state and should persist in such a condition due to heating until the bias current *I* is set to 0. Such evidence suggests that, during a CFE process, $I_{S}$ and $I_{R}$ should always be the same. Since the observed phenomenology is different, we conclude that the hot electron-injection mechanism related to field emission as the predominant cause of the gating effect should be excluded.

### 3.6. Unconventional Sum Rule

**Figure 15:**
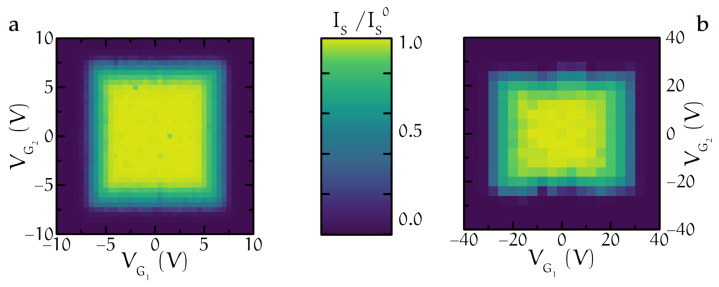
(**a**,**b**) Combined effect of two electric fields on titanium Dayem bridges. Color plot of the normalized switching current as a function of $V_{G_{1}}$ (x-axis) and $V_{G_{2}}$ (y-axis) for two different devices (A and B).

Another strong evidence against a trivial heating or a direct power-injection origin of the supercurrent suppression comes from the evolution of the switching current of a superconducting wire under the action of a pair of lateral side gates. Using a titanium Dayem bridge consisting of double gate-flanked nanoconstrictions interrupting a Ti strip [6], it was possible to assess the mutual influence and the spatial extension of two opposite gate electrodes’ effect on the suppression of the switching current. Figure 15a,b show two contour plots of the normalized switching current $I_{S}/I_{S}^{0}$ as a function of $V_{G_{1}}$ (x-axis) and $V_{G_{2}}$ of two typical devices A and B. The quantitative difference in the values of $V_{G}^{C}$ between the two systems was attributed to the difference in the gate-Dayem bridge (∼80 nm in sample A and ∼120 nm in sample B). The observed square-like shape indicates the existence of a voltage threshold $V_{th}$: when one of the two gates is biased above $V_{th}$, the critical supercurrent is suppressed by a fraction that is not dependent on the voltage applied to the other gate. In other words, the effect of the two gates on $I_{S}$ are independent and no obvious sum rule exists between the actions of the two voltages. Such evidence suggests that the gate-driven suppression of the supercurrent is likely related to a surface effect, which affects non-locally superconductivity, i.e., once the electric field established at one of the surfaces of the superconductor overcomes a critical value, its effect is propagated inside the superconducting body over a distance at least comparable with the device width. In agreement with previous experiments [2] and calculations [64], the surface perturbation could affect the superconductor at a depth of the order of the superconducting coherence length ξ. Furthermore, the aforementioned behavior is hardly comparable with the picture of a direct heat/power injection due to charge transport from/to the gate. Indeed, in the latter case, a sum rule for the total power $P_{sum}$ is expressed as $V_{G_{1}}^{2}/R_{1}+V_{G_{2}}^{2}/R_{2}$, where $R_{1,2}$ are the gate-superconductor leakage resistances for gates 1 and 2, respectively.

## 4. Summary and Further Research

Along with this review, we showed electrostatic control of the superconducting properties in several all-metallic Josephson weak-links: niobium and vanadium Dayem bridges and titanium suspended wires (Section 2). On the one hand, we focused on the technological application of the effect, demonstrating supercurrent suppression on the material that represents the industrial standard for superconducting electronics, niobium. Moreover, the vanadium Dayem bridge experiments showed the potential for electric signal rectification of such geometry. On the other hand, we investigated the dynamic of the phase slip in a titanium Dayem bridge JJ under the effect of an electrostatic field. The results demonstrated that it is impossible to ascribe the modifications of the shape of the SCPDs to a conventional heating effect. In particular, in the framework of the established KFD theory, it was not possible to interpret the width of the distribution with the usual parameters. Furthermore, the experiment carried out with a suspended titanium wire demonstrated that the presence of the substrate is not critical to the occurrence of the effect. Such evidence confutes any possible contribution to superconductivity quenching due to the existence of an injection current that flows in the substrate.

In the second part of this review, we faced the hypothesis of the thermal origin of the electrostatic effect on BCS superconductors. Thanks to finite element simulations performed on a system with the same geometry as the suspended titanium transistor, we demonstrated that cold field emission cannot be a satisfactory explanation for suppression of the supercurrent. Even assuming that single electrons are emitted from the gate and absorbed by the junction, the local increase in the electronic temperature of the system is incompatible with the superconducting state. Additionally, the nontrivial summation rule of two side gates in quenching of the supercurrent is further evidence that a trivial thermal effect is not able to explain our unconventional gating effect.

To shed light on these experiments and to progress towards an understanding of the origin of the effect, a set of complementary experiments are required. For example, SQUID microscopy could provide useful information on the distribution of supercurrents in a field-affected region of the superconductor. Additionally, scanning tunnelling spectroscopy and scanning gate experiments are critical to investigate spatial variation in the superconducting gap. Moreover, radiofrequency-based experiments are crucial to acquire information on the characteristic time scale of the effect and the role of quasiparticle excitation in quenching of the supercurrent.

Moreover, a theoretical model able to explain the observed phenomenology has not been provided yet. From a technological point of view, the unconventional field effect promises to be suitable for a wide range of applications. We already demonstrated that the rectification properties of a Dayem bridge system and more complex devices such as gate-controlled radiation detector [65,66,67], signal routers, and computational systems [25,68] are within reach. Moreover, the superconducting gate-controlled transistors are suitable for high-current application thanks to the highly isolated gate, which is a fundamental requirement for high-power applications such as for power metal–oxide–semiconductor field-effect transistors (MOSFETs) and insulated-gate bipolar transistors (IGBTs).

## Data Availability

Data sharing is not applicable to this article.
